# Supplementary material for: A TonB-dependent transporter is required for secretion of protease PopC across the bacterial outer membrane
Source: Nat Commun. 2019 Mar 25;10:1360. doi: 10.1038/s41467-019-09366-9 (PMC6434023; doi:10.1038/s41467-019-09366-9)
Supplement: Supplementary file 1 — Supplementary Information [file 41467_2019_9366_MOESM1_ESM.docx]

**Supplementary Information for**

**A TonB-dependent transporter is required for secretion of protease PopC across the bacterial outer membrane**

Nuria Gómez-Santos, Timo Glatter, Ralf Koebnik, Magdalena Anna Świątek-Połatyńska,

Lotte Søgaard-Andersen*

**This PDF file includes:**

Supplementary Figs. 1-7

Supplementary Tables 1-4

Supplementary References

**Other Supplementary Materials for this manuscript includes the following:**

Source Data file

**
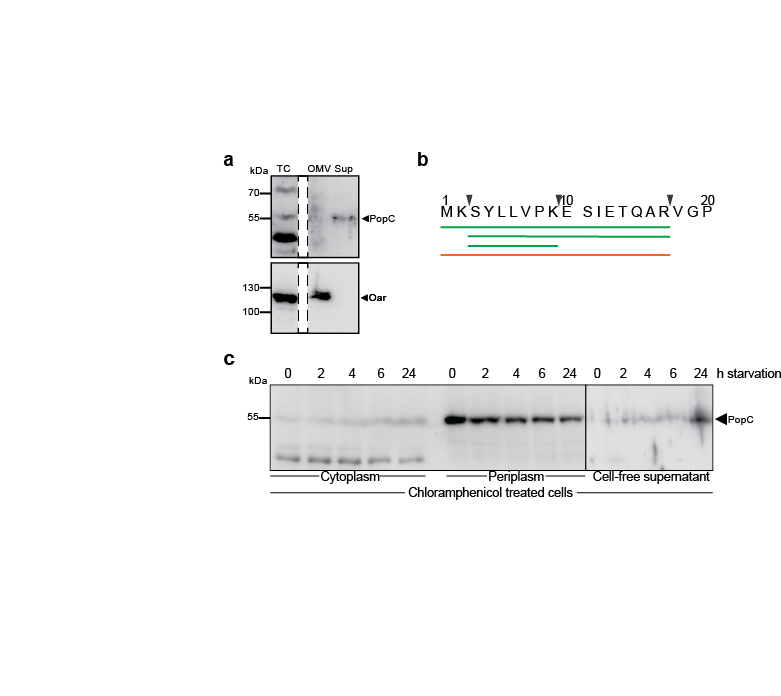
**

**Supplementary Figure 1. PopC is secreted to the extracellular milieu as a full-length protein and is not associated with outer membrane vesicles (OMV). (a)** Immunoblots of total cell extract (TC), OMV and cell-free supernatant (Sup) from 6 h starving WT cells using α-PopC and α-Oar antibodies. White bars between dashed lines indicate lanes that have been removed from the same blot. (**b**) N-terminal PopC peptides detected by mass spectrometry. The 20 N-terminal amino acid residues of native PopC are shown together with trypsin cleavage sites (black arrowheads). N-terminal PopC peptides in total cell extract (green) and cell-free supernatant (orange) detected in 6 h starving WT cells are indicated. (**c**) PopC is secreted to the extracellular milieu from the periplasm. Immunoblots of fractions enriched for cytoplasmic and periplasmic proteins as well as proteins from the cell-free supernatant from starving WT cells treated with chloramphenicol. Source data for S1a, c are provided as a Source Data file.

**
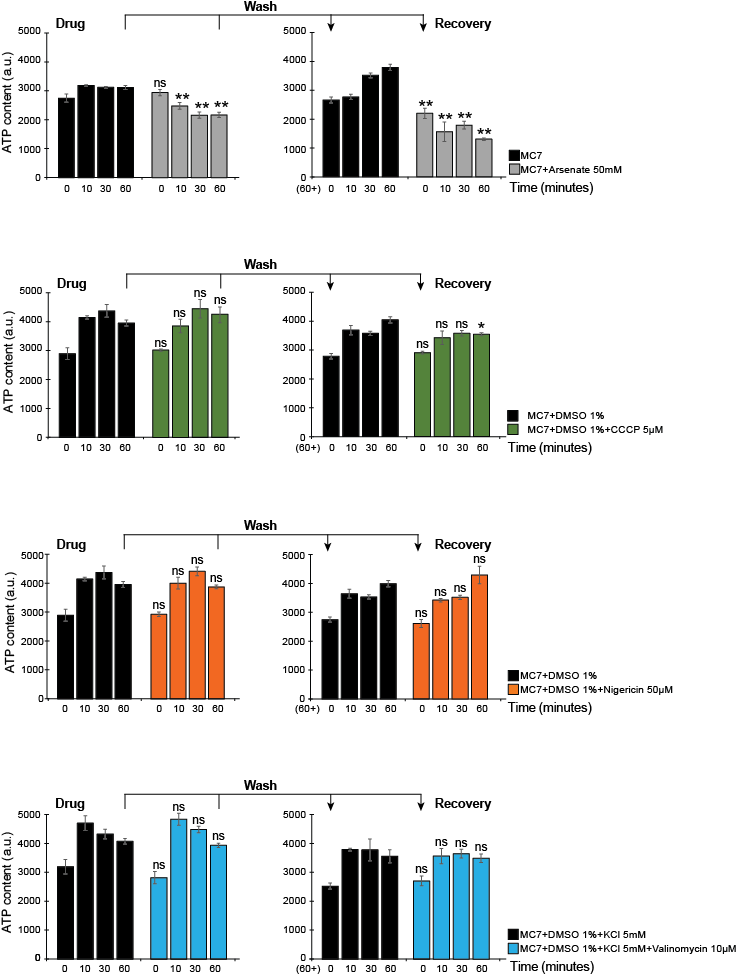
**

**Supplementary Figure 2. ATP content in starving *M. xanthus* cells treated with various drugs.** WT cells were simultaneously exposed to starvation and the indicated drugs. After 60 min, drugs were washed away. At indicated time points, cells were harvested and analysed for ATP content. N=4. Error bars: s.d. At each time point, treated samples are compared to the relevant untreated sample by t-test, *, ** and ns indicate p-value ≤ 0.05, ≤ 0.01 and > 0.05, respectively. Data for MC7 + DMSO in the diagrams for CCCP and nigericin are the same. Source data are provided as a Source Data file.


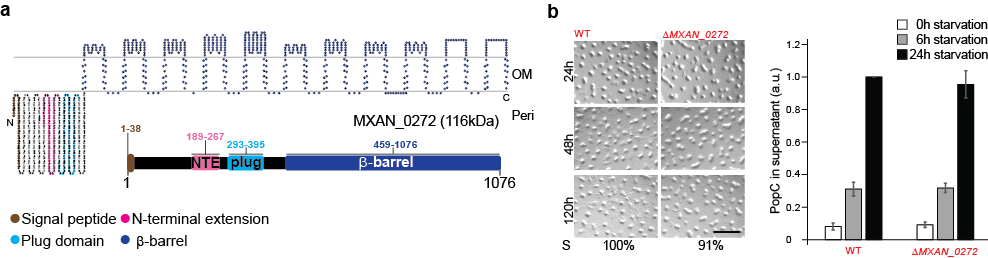


Supplementary Figure 3. Membrane topology and developmental phenotype caused by lack of the TBDT MXAN_0272. (a) Predicted membrane topology and domain structure of MXAN_0272. (b) Developmental phenotype of and PopC secretion by the Δ*MXAN_0272* mutant. Strains were imaged at the indicated time points of starvation. Sporulation (S) is expressed as percentage of WT. Scale bar, 1 mm. Accumulation of PopC in cell-free supernatants of strains of indicated genotype were detected and analyzed as in Fig. 4a. N=3. Source data for S3b are provided as a Source Data file.


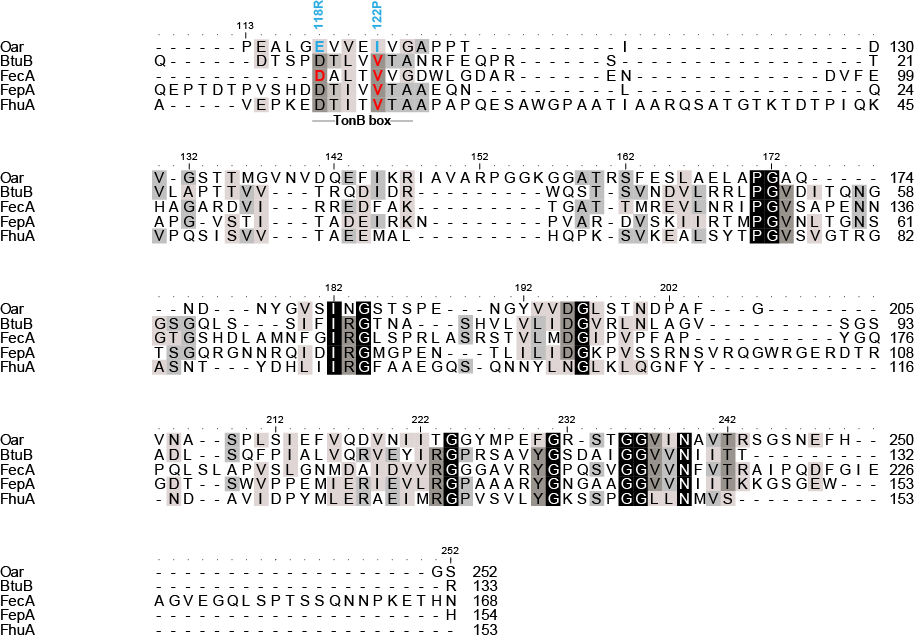


Supplementary Figure 4. Identification of the TonB box in the plug domain of Oar. Alignment of plug domain from *M. xanthus* Oar and *E. coli* TBDTs. The TonB box is indicated. Substitutions of residues marked in red reduce function of the relevant TBDT^1^. Oar residues marked in blue were substituted to the indicated amino acids (see also Fig. 5c). Numbers above the alignment indicate amino acid position using Oar as reference. Alignments where generated with T-coffee^2^ and graphically represented with BioEdit^3^.


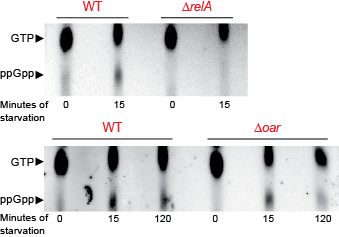


Supplementary Figure 5. Stringent response is induced in Δ*oar* cells. Cells of the indicated genotypes were exposed to starvation in suspension, nucleotides extracted at the indicated time points for each of the indicated strains and separated by thin layer chromatography (Methods). GTP and ppGpp are indicated. The Δ*relA* mutant served as a negative control. Source data are provided as a Source Data file.


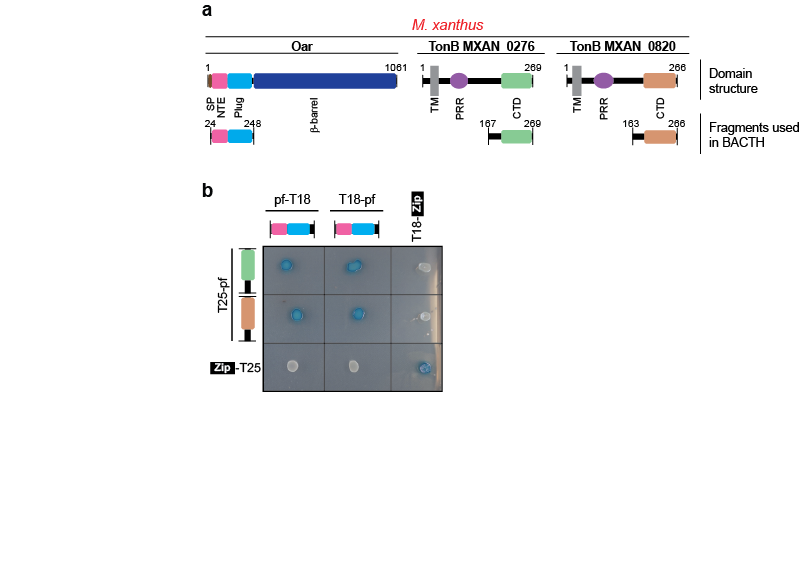


Supplementary Figure 6. Oar N-terminal domains interact with several TonB proteins from *M. xanthus.* (a) Domain structure of Oar, TonB MXAN_0276 and TonB MXAN_0820, and fragments used in BACTH analysis shown as in Fig. 5a. (b) BACTH analysis for interactions between N-terminal domains of Oar and the CTD of TonB MXAN_0276 and TonB MXAN_0820. Results presented and analyzed as in Fig. 5b.


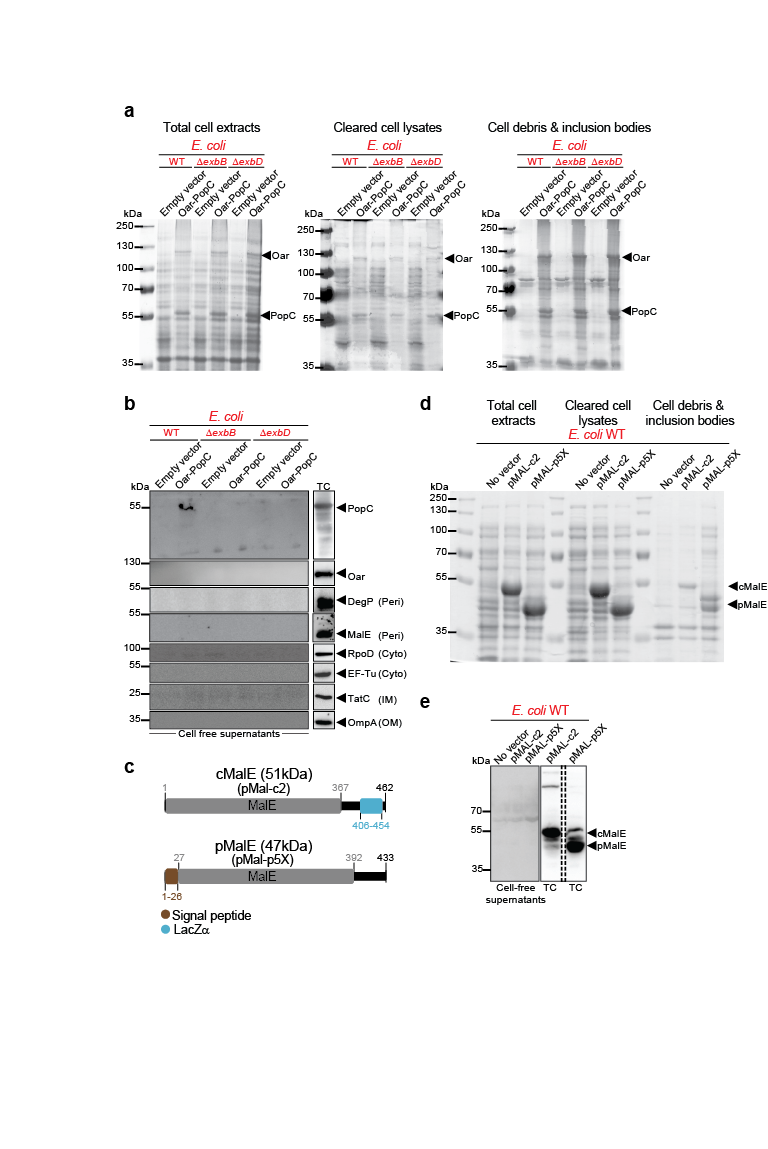


Supplementary Figure 7. Oar- and ExbB/ExbD-dependent PopC secretion by *E. coli.* (a) Oar and PopC co-expression in *E. coli*. Coomassie Brilliant Blue stained SDS-PAGE gels loaded with total cell extracts, cleared cell lysates and cell debris/inclusion bodies after 6 h of induction of the indicated proteins in *E. coli* strains of the indicated genotypes (red). (b) Protein detection by immunoblotting in cell-free supernatants of *E. coli* strains shown in (a). DegP, MalE, RpoD, EF-Tu, TatC and OmpA are markers for the indicated subcellular fractions. TC: total cell extracts from WT cells. (c) Domain architecture of the cytoplasmic MalE-LacZα (cMalE) protein and the periplasmic MalE (pMalE) protein encoded by the plasmid vectors pMal-c2 and pMal-p5x, respectively. Notice that cMalE is fused to LacZα and therefore has a higher molecular weight (MW) than pMalE. MW for pMalE is indicated for the pre-protein including the signal peptide. Processed pMalE has a calculated MW of 45 kDa. (d) cMalE and pMalE expression in *E. coli*. Coomassie Brilliant Blue stained SDS-PAGE gels loaded with total cell extracts, cleared cell lysates and cell debris/inclusion bodies after 6 h of induction in a WT *E. coli* strain containing the indicated plasmid vectors. (e) Protein detection by immunoblotting using α-MalE antibodies of cell-free supernatants and total cell extracts (TC) of *E. coli* strains shown in (d). Source data for S7b, e are provided as a Source Data file.

Supplementary Table 1. Comparison of Oar to TBDTs with available crystal structures

| **Protein^1^** | **Length**  **(aa)** | **β-strands** | **% identity^3^** | **Confidence^4^** | **Reference** |
| --- | --- | --- | --- | --- | --- |
| Oar (*Mxan*) | 1061 | 22^2^ | NA | NA | NA |
| PirA (*Paer*) | 742 | 22 | 18 | 100 | ^4^ |
| CirA (*Ecol*) | 663 | 22 | 17 | 100 | ^5^ |
| FptA (*Paer*) | 720 | 22 | 17 | 100 | ^6^ |
| FyuA (*Ypes*) | 673 | 22 | 17 | 100 | ^7^ |
| BtuB (*Ecol*) | 614 | 22 | 16 | 100 | ^8-10^ |
| FhuA (*Ecol*) | 747 | 22 | 16 | 100 | ^11,12^ |
| FauA (*Bper*) | 734 | 22 | 16 | 100 | ^13^ |
| ShuA (*Sdys*) | 660 | 22 | 16 | 100 | ^14^ |
| HasR (*Smar*) | 899 | 22 | 16 | 100 | ^15^ |
| FpvAI (*Paer*) | 815 | 22 | 15 | 100 | ^16-19^ |
| PiuA (*Paer*) | 753 | 22 | 15 | 100 | ^4^ |
| FecA (*Ecol*) | 774 | 22 | 14 | 100 | ^20,21^ |
| FepA (*Ecol*) | 746 | 22 | 14 | 100 | ^22^ |
| PirA (*Abau*) | 743 | 22 | 14 | 100 | ^4^ |
| PiuA (*Abau*) | 743 | 22 | 14 | 100 | ^4^ |
| FusA (*Patr*) | 704 | 22 | 14 | 100 | ^23^ |
| FetA (*Pflu*) | 743 | 22 | 13 | 100 | ^24^ |
| FrpB (*Nmen*) | 692 | 22 | 12 | 100 | ^25^ |
| ZnuD (*Nmen*) | 748 | 22 | 12 | 100 | ^26^ |
| TbpA (*Nmen*) | 913 | 22 | 11 | 100 | ^27^ |
| BT1763 (*Bthe*) | 1041 | 22 | 11 | 100 | ^28^ |

^1^ Organisms: *Mxan*: *Myxococcus xanthus*; *Paer*: *Pseudomonas aeruginosa*; *Ecol*: *Escherichia coli*; *Ypes*: *Yersinia pestis*; *Bper*: *Bordetella pertussis*; *Sdys*: *Shigella dysenteriae*; *Smar*: *Serratia marcescens*; *Abau*: *Acinetobacter baumannii*; *Patr*: *Pectobacterium atrosepticum*; *Pflu*: *Pseudomonas fluorescens*; *Nmen*: *Neisseria meningitidis*; *Bthe*: *Bacteroides thetaiotaomicron*.

^2^ Number of transmembrane β-strands in Oar were predicted by BOCTOPUS.

^3^ % identity between Oar and indicated protein. Values were calculated using Phyre2^29^. NA: not applicable.

^4^ Confidence values were calculated using Phyre2^29^. Values represent the probability (from 0 to 100) that the match between Oar and a given template is a true homology. NA: not applicable.

Supplementary Table 2. *M. xanthus* and *E. coli* strains and plasmids used in this study

| **Strain** | **Description^1^** | **Reference or source** |
| --- | --- | --- |
| ***M. xanthus*** |  |  |
| DK101 (WT) | *pilQ1* | ^30^ |
| DK10410  DK1217  MS1000 | Δ*pilA*  *aglB* (*= aglQ*)  *pilQ1*, Δ*relA* | ^31^  ^32,33^  ^34^ |
| SA3442 | *pilQ1*, *popD*::*aadA* (Str^r^) | ^35^ |
| SA3465 | *pilQ1*, *popC*::*aadA*(Str^r^) | ^35^ |
| SA6695 | *pilQ1*, Δ*MXAN_0272* | This study |
| SA5544 | *pilQ1*, Δ*oar* | This study |
| SA5564 | *pilQ1*, Δ*oar* / P*_pilA_* - *oar* (pNG013) (Km^r^) | This study |
| SA6650 | *pilQ1*, Δ*csgA* | This study |
| SA5555 | *pilQ1*, *popD::aadA* Δ*oar* (Str^r^) | This study |
| SA7946 | *pilQ1*, *popC::aadA* Δ*oar* (Str^r^) | This study |
| SA5594 | *pilQ1*, Δ*oar* Δ*csgA* | This study |
| SA6605 | *pilQ1*, Δ*ton^oar^* | This study |
| SA6676 | *pilQ1*, Δ*ton^oar^* Δ*oar* | This study |
| SA5591 | *pilQ1* / P*_pilA_*- *csgA* (pNG255) (Km^r^) | This study |
| SA7948 | *pilQ1*, *popC::aadA* / P*_pilA_* - *csgA* (pNG255) (Str^r^Km^r^) | This study |
| SA6612 | *pilQ1*, *popD::aadA* / P*_pilA_ - csgA* (pNG255) (Str^r^Km^r^) | This study |
| SA5595 | *pilQ1*, Δ*oar* / P*_pilA_* - *csgA* (pNG255) (Km^r^) | This study |
| SA6613 | *pilQ1*, *popD::aadA* Δ*oar* / P*_pilA_* - *csgA* (pNG255) (Str^r^Km^r^) | This study |
| SA6667 | *pilQ1*, Δ*ton^oar^* / P*_pilA_* - *csgA* (pNG255) (Km^r^) | This study |
| SA7949 | *pilQ1*, Δ*ton^oar^* Δ*oar* / P*_pilA_* - *csgA* (pNG255) (Km^r^) | This study |
| SA7937 | *pilQ1*, *oar*^Δ^*^NTE^*^Δ^*^plug^* | This study |
| SA7938 | *pilQ1*, *oar*^Δ^*^NTE^* | This study |
| SA7939 | *pilQ1*, *oar*^Δ^*^plug^* | This study |
| SA7957 | *pilQ1*, Δ*oar* / P*_pilA_* - *oar^E118R^* (pNG140) (Km^r^) | This study |
| SA7959 | *pilQ1*, Δ*oar* / P*_pilA_* - *oar^I122P^* (pNG142) (Km^r^) | This study |
| SA5597 | *pilQ1*, Δ*tonB1* | This study |
| SA5596 | *pilQ1*, Δ*exbB1* | This study |
| SA6610 | *pilQ1*, Δ*exbD1* | This study |
| SA5571 | *pilQ1*, Δ*exbD2* | This study |
| SA6615 | *pilQ1*, Δ*tonB1* / P*_pilA_* - *tonB1* (pNG025) (Km^r^) | This study |
| SA6659 | *pilQ1*, Δ*exbB1* / P*_pilA_* - *exbB1* (pNG032) (Km^r^) | This study |
| SA6658 | *pilQ1*, Δ*exbD1* / P*_pilA_* - *exbD1* (pNG031) (Km^r^) | This study |
| SA6657 | *pilQ1*, Δ*exbD2* / P*_pilA_* - *exbD2* (pNG030) (Km^r^) | This study |
| SA6614 | *pilQ1*, Δ*tonB1* / P*_pilA_* - *oar* (pNG013) (Km^r^) | This study |
| ***E. coli*** |  |  |
| Mach1 | Δ*recA*1398 *endA*1 *tonA* Φ80Δ*lacM*15 Δ*lacX*74 *hsdR*(r_K_^-^ m_K_^+^) | Invitrogen |
| Rosetta-2(DE3) | F- *ompT hsdSB*(rB^-^ mB^-^) *gal dcm* (DE3) pRARE2 (Cam^r^) | Novagen |
| SA6683 | F- *ompT hsdSB*(rB^-^ mB^-^) *gal dcm* (DE3) pRARE2 (Cam^r^) Δ*tonB* | This study |
| SA7953 | F- *ompT hsdSB*(rB^-^ mB^-^) *gal dcm* (DE3) pRARE2 (Cam^r^) Δ*exbB* | This study |
| SA7954 | F- *ompT hsdSB*(rB^-^ mB^-^) *gal dcm* (DE3) pRARE2 (Cam^r^) Δ*exbD* | This study |
| BTH101 | F- *cya*-99 *araD*139 *galE*15 *galK*16 *rpsL1* (Str^r^) *hsdR*2 *mcrA*1 *mcrB*1 | Euromedex |
| JW5195 | *tonB* (F-, Δ(*araD*-*araB*)567, Δ*lacZ4787*(::rrnB-3), λ-, Δ*tonB760*::*kan*, *rph-1*, Δ(*rhaD*-*rhaB*)*568*, *hsdR514*) | Dharmacon |
| JW2974 | *exbB* (F-, Δ(*araD*-*araB*)567, Δ*lacZ4787*(::rrnB-3), λ-, Δ*exbB779*::*kan*, *rph-1*, Δ(*rhaD*-*rhaB*)*568*, *hsdR514*) | Dharmacon |
| JW2973 | *exbD* (F-, Δ(*araD*-*araB*)567, Δ*lacZ4787*(::rrnB-3), λ-, Δ*exbD778*::*kan*, *rph-1*, Δ(*rhaD*-*rhaB*)*568*, *hsdR514*) | Dharmacon |
| JW0157 | *degP* (F-, Δ(*araD*-*araB*)567, Δ*lacZ4787*(::rrnB-3), λ-, Δ*degP775*::*kan*, *rph-1*, Δ(*rhaD*-*rhaB*)*568*, *hsdR514*) | Dharmacon |
| JW3994 | *malE* (F-, Δ(*araD*-*araB*)567, Δ*lacZ4787*(::rrnB-3), λ-, Δ*malE730*::*kan*, *rph-1*, Δ(*rhaD*-*rhaB*)*568*, *hsdR514*) | Dharmacon |
| JW0940 | *ompA* (F-, Δ(*araD*-*araB*)567, Δ*lacZ4787*(::rrnB-3), λ-, Δ*ompA772*::*kan*, *rph-1*, Δ(*rhaD*-*rhaB*)*568*, *hsdR514*) | Dharmacon |
| JW0052 | *surA* (F-, Δ(*araD*-*araB*)567, Δ*lacZ4787*(::rrnB-3), λ-, Δ*surA765*::*kan*, *rph-1*, Δ(*rhaD*-*rhaB*)*568*, *hsdR514*) | Dharmacon |
| JW3815 | *tatC* (F-, Δ(*araD*-*araB*)567, Δ*lacZ4787*(::rrnB-3), λ-, Δ*tatC781*::*kan*, *rph-1*, Δ(*rhaD*-*rhaB*)*568*, *hsdR514*) | Dharmacon |
| JW3301 | *tufA* (F-, Δ(*araD*-*araB*)567, Δ*lacZ4787*(::rrnB-3), λ-, Δ*tufA749*::*kan*, *rph-1*, Δ(*rhaD*-*rhaB*)*568*, *hsdR514*) | Dharmacon |
| JW3943 | *tufB* (F-, Δ(*araD*-*araB*)567, Δ*lacZ4787*(::rrnB-3), λ-, Δ*tufB756*::*kan*, *rph-1*, Δ(*rhaD*-*rhaB*)*568*, *hsdR514*) | Dharmacon |
| **Plasmids** | **Description^2^** | **Reference or source** |
| pBJ114 | Km^r^ *galK* | ^36^ |
| pSW105 | P*_pilA_* Km^r^ | ^37^ |
| pNG058 | pBJ114, in-frame deletion construct for *MXAN_0272* Km^r^ | This study |
| pNG059 | pBJ114, in-frame deletion construct for *MXAN_0821* Km*^r^* | This study |
| pNG020 | pBJ114, in-frame deletion construct for *MXAN_1450* (*oar*) Km^r^ | This study |
| pSD12 | pBJ114, in-frame deletion construct for *MXAN_1294* (*csgA*) Km^r^ | This study |
| pNG014 | pBJ114, in-frame deletion construct for *MXAN_1449* (*tonB1*) Km^r^ | This study |
| pNG015 | pBJ114, in-frame deletion construct for *MXAN_1448* (*exbB1*) Km^r^ | This study |
| pNG016 | pBJ114, in-frame deletion construct for *MXAN_1446* (*exbD2*) Km^r^ | This study |
| pNG017 | pBJ114, in-frame deletion construct for *MXAN_1447* (*exbD1*) Km^r^ | This study |
| pNG018 | pBJ114, in-frame deletion construct for *MXAN_1446-1449* (*ton^oar^*) Km^r^ | This study |
| pNG084 | pBJ114, in-frame deletion construct for *oar*^Δ^*^NTE^*^Δ^*^plug^* Km^r^ | This study |
| pNG085 | pBJ114, in-frame deletion construct for *oar*^Δ^*^NTE^* Km^r^ | This study |
| pNG086 | pBJ114, in-frame deletion construct for *oar*^Δ^*^plug^* Km^r^ | This study |
| pNG013 | pSW105 P*_pilA_* - *oar* Km^r^ | This study |
| pNG140 | pSW105 P*_pilA_* - *oar^E118R^* Km^r^ | This study |
| pNG142 | pSW105 P*_pilA_* - *oar^I122P^* Km^r^ | This study |
| pNG255 | pSW105 P*_pilA_* - *csgA* Km^r^ | This study |
| pNG025 | pSW105 P*_pilA_* - *tonB1* Km^r^ | This study |
| pNG030 | pSW105 P*_pilA_* - *exbD2* Km^r^ | This study |
| pNG031 | pSW105 P*_pilA_* - *exbD1* Km^r^ | This study |
| pNG032 | pSW105 P*_pilA_* - *exbB1* Km^r^ | This study |
| pKT25 | Two-hybrid plasmid, *cyaAT25* C-terminal fusion Km^r^ | ^38^(Euromedex) |
| pUT18 | Two-hybrid plasmid, *cyaAT18* N-terminal fusion Amp^r^ | ^38^(Euromedex) |
| pKNT25 | Two-hybrid plasmid, *cyaAT25* N-terminal fusion Km^r^ | ^39^(Euromedex) |
| pUT18C | Two-hybrid plasmid, *cyaAT18* C-terminal fusion Amp^r^ | ^38^(Euromedex) |
| pKT25-zip | Two-hybrid control plasmid Km^r^ | D. Ladant (Euromedex) |
| pUT18C-zip | Two-hybrid control plasmid Amp^r^ | D. Ladant (Euromedex) |
| pNG040 | pUT18, Oar_24-248_ - *cyaT18* Amp^r^ | This study |
| pNG042 | pKT25, cyaT25 - Oar_24-248_  Km^r^ | This study |
| pNG044 | pKT25, cyaT25 - _Mx_TonB1_147-250_ Km^r^ | This study |
| pNG045 | pKNT25, Oar_24-248_ - *cyaT25* Km^r^ | This study |
| pNG047 | pKNT25, _Mx_TonB1_147-250_ - *cyaT25* Km^r^ | This study |
| pNG048 | pUT18, _Mx_TonB1_147-250_ - *cyaT18* Amp^r^ | This study |
| pNG049 | pUT18C, *cyaT18* - Oar_24-248_ Amp^r^ | This study |
| pNG051 | pUT18C, *cyaT18* - _Mx_TonB1_147-250_ Amp^r^ | This study |
| pNG090 | pUT18C, *cyaT18* - _Ec_TonB_139-239_ Amp^r^ | This study |
| pNG091 | pUT18, _Ec_TonB_139-239_ - *cyaT18* Amp^r^ | This study |
| pNG092 | pKT25, *cya*T25 - _Ec_TonB_139-239_ Km^r^ | This study |
| pNG093 | pKNT25, _Ec_TonB_139-239_ - *cyaT25* Km^r^ | This study |
| pNGMP2 | pKT25, *cya*T25 - TonB MXAN_0276_167-269_ Km^r^ | This study |
| pNGMP6 | pKT25, *cya*T25 - TonB MXAN_0820_163-266_ Km^r^ | This study |
| pRSFDuet-1 | Duet vector (double T7 promoters) RSF1030 ori Km^r^ | Novagen |
| pNG026 | pRSFDuet-1, *oar* Km^r^ | This study |
| pNG027 | pRSFDuet-1, *popC* Km^r^ | This study |
| pNG028 | pRSFDuet-1, *oar popC* Km^r^ | This study |
| pNG088 | pRSFDuet-1, *MXAN_0272* Km^r^ | This study |
| pNG089 | pRSFDuet-1, *MXAN_0272 popC* Km^r^ | This study |
| pMal-c2 | Amp^r^, cytoplasmic MalE | New England Biolabs |
| pMal-p5X | Amp^r^, periplasmic MalE | New England Biolabs |

^1^ Plasmids for ectopic expression of genes in *M. xanthus* are all derivatives of pSW105 and were integrated by site specific recombination at the chromosomal *attB* site. Plasmids used for complementation are listed in parentheses. P*_pilA_* indicates that these genes were expressed from the *pilA* promoter.

**Table S3.** Oligonucleotides used in this study^1^

| **Primer name** | **Sequence 5'- 3'** | **Brief description** |
| --- | --- | --- |
| NG004 | CACCgagctcAGCCCTGGCTGACGCGCGCC | csgA under p*PilA* Rv |
| NG005 | CCCaagcttGTCGACCTACCAGGGCACTTCGGTCC | csgA under p*PilA* Fw |
| NG112 | GCCGgaattcACACCACCGACCACGCCA | Oar mutant (A EcoRI) |
| NG157 | GCGGACCTGGGTTTCCCGGAGCACTCG | Oar mutant (B) |
| NG158 | CGGGAAACCCAGGTCCGCTTCGGCATC | Oar mutant (C) |
| NG115 | GGTTggatccGGACCCCACACCGCAGTT | Oar mutant (D BamHI) |
| NG116 | GGCTTGATGATCCGGCAG | Oar mutant (E) |
| NG117 | GGTCGGCTGGCACACCAC | Oar mutant (F) |
| NG118 | GAGCAGACGGTCGTCACC | Oar mutant (G) |
| NG119 | TGGAACTCGTTGGAGCCC | Oar mutant (H) |
| NG125 | GGGGtctagaATGCACTTGAACCGAGTGCT | Oar complementation under p*PilA*Fw |
| NG126 | GGGCaagcttTCAGAACGTGTACCGGATGC | Oar complementation Rv |
| NG176 | TCTCgaattcCTCACCGAGCGCCTCGGG | MXAN_1449 mutant (A EcoRI) |
| NG177 | CAGCTTGAAGACTGAATC GAACATGCACTA | MXAN_1449 mutant (B) |
| NG178 | GATTCAGTCTTCAAGCTG CCGCGCTGAGTC | MXAN_1449 mutant (C) |
| NG179 | GGCCGaagcttTGAGCGCCTCGGCGATAC | MXAN_1449 mutant (D HindIII) |
| NG180 | AAGACgaattcGAAGAAGCCCAAGAAGCC | MXAN_1448 mutant (A EcoRI) |
| NG181 | GTTCGAGGAAGAGTGAATTGCATGGTTGGG | MXAN_1448 mutant (B) |
| NG182 | ATTCACTCTTCCTCGAACGCCGCCTGAGCC | MXAN_1448 mutant (C) |
| NG183 | AGTTCaagcttGCCTGGGGCTTGACCCAC | MXAN_1448 mutant (D HindIII) |
| NG184 | CTGGGgaattcTGTGGGCCACCTGGGCCG | MXAN_1447 mutant (A EcoRI) |
| NG185 | CTCCTCGACTGCTGACATTCCCATGTCTTT | MXAN_1447 mutant (B) |
| NG186 | ATGTCAGCAGTCGAGGAGAAGAAGTAATGG | MXAN_1447 mutant (C) |
| NG187 | AAGCTaagcttCCATGAGGAGTGAACTCT | MXAN_1447 mutant (D HindIII) |
| NG188 | GGACGgaattcCCCGCCACCTGGGTGGTG | MXAN_1446 mutant (A EcoRI) |
| NG189 | GGGTGCGGGCTTGCGGCCGGCCATTACTTC | MXAN_1446 mutant (B) |
| NG190 | GGCCGCAAGCCCGCACCCACGCCCTGAAAC | MXAN_1446 mutant (C) |
| NG191 | TGGCCaagcttGCCGGACATCTCGATGAC | MXAN_1446 mutant (D HindIII) |
| NG192 | GCGCAACGGCGATGCGCT | MXAN_1449 mutant (E) |
| NG193 | GACTCGGAGATGTCCACC | MXAN_1449 mutant (F) |
| NG194 | CTCCATCTGGCTGTCGAC | MXAN_1449 mutant (G) |
| NG195 | CCGCCAATCACACCACCG | MXAN_1449 mutant (H) |
| NG196 | AGGAGATTGAGGTCACGC | MXAN_1448 mutant (E) |
| NG197 | GATGTCCTTCTCGAGGAG | MXAN_1448 mutant (F) |
| NG198 | CTGGCCAAAGGCGACCTG | MXAN_1448 mutant (G) |
| NG199 | TGTCCACCGAGCGCGCGT | MXAN_1448 mutant (H) |
| NG200 | GCAAGACGCGGTCTGACA | MXAN_1447 mutant (E) |
| NG201 | GCCTGCCGCAGGCGTGCC | MXAN_1447 mutant (F) |
| NG202 | GAGCGACATCAACGTCAC | MXAN_1447 mutant (G) |
| NG203 | GGACTTGCGGGCCACGTC | MXAN_1447 mutant (H) |
| NG204 | GCCATCCCCGCGGTGATG | MXAN_1446 mutant (E) |
| NG205 | CTGACCTCCTGGCGGCCT | MXAN_1446 mutant (F) |
| NG206 | AAGCCCCAGGCGCAGCCG | MXAN_1446 mutant (G) |
| NG207 | AGTGCCCTGGATGATGGC | MXAN_1446 mutant (H) |
| NG208 | GAGTCtctagaATGTTCGATTCAGTCCTT | MXAN_1449 complementation under pPilAFw |
| NG209 | CGCGCaagcttTCAGCGCGGCAGCTTGAA | MXAN_1449 complementation Rv |
| NG210 | TCAACtctagaATGCAATTCACTCTCGCA | MXAN_1448 complementation under pPilAFw |
| NG211 | CCCGGaagcttTCAGGCGGCGTTCGAGGA | MXAN_1448 complementation Rv |
| NG212 | CAGGTtctagaATGGGAATGTCAGCAGGC | MXAN_1447 complementation under pPilAFw |
| NG213 | GCGCGaagcttTTACTTCTTCTCCTCGAC | MXAN_1447 complementation Rv |
| NG214 | AGAAGtctagaATGGCCGGCCGCAAGCAA | MXAN_1446 complementation under pPilAFw |
| NG215 | GAAGAaagcttTCAGGGCGTGGGTGCGGG | MXAN_1446 complementation Rv |
| NG220 | GGGTGCGGGGACTGAATCGAACATGCACTA | MXAN_1446-1449 mutant (B) |
| NG221 | GATTCAGTCCCCGCACCCACGCCCTGAAAC | MXAN_1446-1449 mutant (C) |
| NG244 | CGAGCGCGTCGATGAGCA | CsgA mutant (E) |
| NG245 | GTAGCGCGGGGTGATGAC | CsgA mutant (F) |
| NG246 | CAACCGCCTGCGCATCCA | CsgA mutant (G) |
| NG247 | GCTGTGCTCCGGGTTCAG | CsgA mutant (H) |
| NG272 | GCACAcatatgAAGTCCTACCTGTTG | PopC Nde duet |
| NG274 | GTCTGCCATGGCGCACTTGAACCGAGTGCTC | Oar Nco duet |
| NG275 | GGGCgaattcTTAGAACGTGTACCGGAT | Oar EcoRI duet |
| NG320 | ATTGCggatccCTTCGCGCAGTCGAGCACG | OarA24E248 BamHI Fw BTH |
| NG321 | AATGCgaattcTCA CTCGTTGGAGCCCGACCG | OarA24E248 EcoRIStop Rv BTH |
| NG322 | ATTGCaagcttGTTCGCGCAGTCGAGCACG | OarA24E248 HindIII Fw BTH |
| NG323 | AATGCgaattcGACTCGTTGGAGCCCGACCG | OarA24E248 EcoRI Rv BTH |
| NG325 | ATTGCggatccCGGCCAGCTTGGCGGGACG | MXAN_1449 G147R250 BamHI Fw BTH |
| NG326 | AATGCgaattcTCAGCGCGGCAGCTTGAA | MXAN_1449 G147R250 EcoRI Stop Rv BTH |
| NG328 | ATTGCaagcttG GGCCAGCTTGGCGGGACG | MXAN_1449 G147R250 HindIII Fw BTH |
| NG329 | AATGCgaattcGAGCGCGGCAGCTTGAAGTT | MXAN_1449 G147R250 EcoRI Rv BTH |
| NG330 | TCCGcaattgTCACCTGAATACGGCCAG | PopC MfeI duet |
| K1 | CAGTCATAGCCGAATAGCCT | k1 (km cassette Fw Keio collection) |
| K2 | CGGTGCCCTGAATGAACTGC | k2 (km cassette Rv Keio collection) |
| NG339 | GGGCACAACTCCTTGTTC | U primer (upstream *tonB* Fw) |
| NG340 | TGTGGTGTGCTGCTATGC | D primer (downstream *tonB* Rv) |
| NG341 | CGGAGCCGGTGGTAGAGC | I-1 primer (inside *tonB* Fw) |
| NG342 | TTAACCTGCCCTTCAATG | I-2 primer (inside *tonB* Rv) |
| NG343 | ACGGCaagcttCGTGGTGCTCAAGGCCGA | MXAN_0272 mutant (A HindIII) |
| NG344 | CCAGCCCAGGAACGCGACACGCATGGC | MXAN_0272 mutant (B) |
| NG345 | GTCGCGTTCCTGGGCTGGTCCCTCTAA | MXAN_0272 mutant (C) |
| NG346 | AGCGCgaattcATGTAGTCCAGGTCGGTG | MXAN_0272 mutant (D EcoRI) |
| NG347 | ATGTCggatccGCCTCCGCCGCCCGTGGA | MXAN_0821 mutant (A BamHI) |
| NG348 | TCCTTTCACGGCGTGTCCAAGCATACG | MXAN_0821 mutant (B) |
| NG349 | GGACACGCCGTGAAAGGAAGCTTCTGA | MXAN_0821 mutant (C) |
| NG350 | AGCGCgaattcCCACGAAGGGCCCGGCGC | MXAN_0821 mutant (D EcoRI) |
| NG356 | AATGCgaattcTTACTGAATTTCGGTGGT | E.coli TonBR Stop EcoRI BTH |
| NG357 | AATGCgaattcGACTGAATTTCGGTGGTGCC | E.coli TonBR EcoRI BTH |
| NG363 | ACGGCGACGAACCCGGAC | MXAN_0272 mutant (E) |
| NG364 | CGATGAAGGTCGCGTTCC | MXAN_0272 mutant (F) |
| NG365 | CCCAACATGGGCCTGGGC | MXAN_0272 mutant (G) |
| NG366 | AGCAGGTAGCGGAAGCGG | MXAN_0272 mutant (H) |
| NG367 | CCGAGCCGCCGAAGCCGG | MXAN_0821 mutant (E) |
| NG368 | CCCAGCGTCGCGTGCCAC | MXAN_0821 mutant (F) |
| NG369 | CGGCAGCCGTTCGCGCGT | MXAN_0821 mutant (G) |
| NG370 | GTCCGAGGCGCCCTTGCG | MXAN_0821 mutant (H) |
| NG371 | GGTTCCCGTCCCGCCAAG | ΔNTE-plug (E) |
| NG372 | CCGTAGTTCGAACCCTCC | ΔNTE-Plug (F) |
| NG373 | CGTCGTTGTGACCGCCAC | ΔNTE-Plug (G) |
| NG374 | CCAGTTCGCGAACACGGA | ΔNTE-Plug (H) |
| NG405 | GCTAAgaattcTGAAAAGCCCGAGGCGCGCTCGTC | MXAN_0821 Insertion Fw EcoRI |
| NG406 | GCTAAaagcttTCACACGCGCGAACGGCTGCCGCG | MXAN_0821 Insertion Rv HindIII |
| NG411 | GGCAATgaattcCTCCTTCGGAATCTCCTT | ΔNTE and ΔNTE-Plug (A EcoRI) |
| NG412 | GGTGCCGCAGCCGCGGATCCGTACAG | ΔNTE-Plug (B) |
| NG413 | TCCGCGGCTGCGGTCACCCGGTCGGGC | ΔNTE-Plug (C) |
| NG414 | GGCAATaagcttATCAACGAGGACCTTCTT | ΔNTE-Plug and Plug (D HindIII) |
| NG415 | CGCCTCGGGAGCCGCGGATCCGTACAG | ΔNTE (B) |
| NG416 | TCCGCGGCTCCCGAGGCGCTCGGTGAG | ΔNTE (C) |
| NG417 | GGCAATaagcttGGTGAACGACGGCGCGAA | ΔNTE (D HindIII) |
| NG418 | GGTGACCGCGAGCAGCTCCACGTTGAC | ΔPlug (B) |
| NG419 | GGCAATgaattcCTGACCACGGTCAAGGAC | ΔPlug (A EcoRI) |
| NG420 | GAGCTGCTCGCGGTCACCCGGTCGGGC | ΔPlug (C) |
| NG421 | CAGGCCCGTGCGATCGCC | ΔNTE-Plug (F) |
| NG422 | CACGCCGCCCTCGACGAC | ΔNTE-plug (E) |
| NG423 | GGCCGAACTCCGGCATGT | ΔNTE-Plug (H) |
| NG426 | GGCAAacatgtCTCGTGTCGCGTTCTCT | 0272 pRSFDuet 1 PciI |
| NG427 | GGCAAgaattcTTAGAGGGACCAGCCCAG | 0272 pRSFDuet 1 EcoRI |
| NG430 | ATTGCggatccCAGTACAGCAACGGCTGCA | E.coli TonBF BamHI BTH |
| NG463 | TGTTTTTATACGTCTGGA | U primer (upstream *exbB* Fw) |
| NG464 | GTTACCGATAAACATCGA | D primer (downstream *exbB* Rv) |
| NG465 | CTGCTGGCTGAAGCGCGT | I-1 primer (inside *exbB* Fw) |
| NG466 | GAGGCCGATTGCCGTTGC | I-2 primer (inside *exbB* Rv) |
| NG467 | AGTCGTTGCGCCGGGTAT | U primer (upstream *exbD* Fw) |
| NG468 | GCATCATTGTGAATTCAC | D primer (downstream *exbD* Rv) |
| NG469 | GCGACGGTAGATGTGAAG | I-1 primer (inside *exbD* Fw) |
| NG470 | GATGGTGGTGTCTTTCTT | I-2 primer (inside *exbD* Rv) |
| NG477 | GGTGGTTGAGATCGTCGGCGCGCCCCCG | Oar plug E118R |
| NG478 | CTACCGAGCGCCTCGGGGAGCAGCTCCACGTTGAC |  |
| NG479 | TGAGATCGTCGGCGCGCCCCCGACCATC | Oar plug V120P |
| NG480 | GGCACCTCACCGAGCGCCTCGGGGAGCAG |  |
| NG481 | CGTCGGCGCGCCCCCGACCATCGACGTG | Oar plug I122P |
| NG482 | GGCTCAACCACCTCACCGAGCGCCTCGGG |  |
| NG483 | TGTACggatccGCGGCTT | Fw BamHI NTE Oar |
| NG484 | TTGCGggtaccCTCGAGG | Rv KpnI plug Oar |
| NG485 | CTCAACCAC**cct**ACCGAGCGC | E118R Rv |
| NG486 | GCGCTCGGT**agg**GTGGTTGAG | E118R Fw |
| NG487 | GACGATCTC**agg**CACCTCACC | V120P Rv |
| NG488 | GGTGAGGTG**cct**GAGATCGTC | V120P Fw |
| NG489 | CGCGCCGAC**ggg**CTCAACCAC | I122P Rv |
| NG490 | GTGGTTGAG**ccc**GTCGGCGCG | I122P Fw |

^1^ Restriction sites are indicated in lowercase and mutations introduced by site directed mutagenesis in bold lowercase.

**Supplementary Table 4.** Species and strains used to generate phylogenetic tree representing the distribution of TBDTs according to domain architecture in different phyla

| **Phylum** | **Representative strain** | **RDP identifier** |
| --- | --- | --- |
| Gemmatimonadetes | *Gemmatimonas aurantiaca* T-27 | S002290711 |
| Calditrichaeota | *Caldithrix abyssi* DSM 13497 | S004069663 |
| Bacteroidetes | *Bacteroides fragilis* ATCC 25285 | S004062379 |
| Ignavibacteriae | *Ignavibacterium album* JCM 16511 | S004062564 |
| Chlorobi | *Chlorobaculum tepidum* TLS | S004064585 |
| Armatimonadetes | *Chthonomonas calidirosea* T49 | S004067624 |
| Cyanobacteria | *Nostoc punctiforme* PCC 73102 | S001094662 |
| Actinobacteria | *Mumia flava* MUSC 201 | S003804991 |
| Thermodesulfobacteria | *Thermodesulfatator indicus* DSM 15286 | S004062585 |
| Aquificae | *Sulfurihydrogenibium azorense* Az-Fu1 | S004062452 |
| Chrysiogenetes | *Desulfurispirillum indicum* S5 | S004067480 |
| Deferribacteres | *Denitrovibrio acetiphilus* DSM 12809 | S004064570 |
| Fibrobacteres | *Chitinivibrio alkaliphilus* ACht1 | S004474191 |
| Firmicutes | *Veillonella parvula* DSM 2008 | S004064250 |
| Fusobacteria | *Fusobacterium nucleatum* ATCC 25586 | S004064231 |
| Planctomycetes | *Blastopirellula marina* DSM 3645 | S003288169 |
| Verrucomicrobia | *Opitutus terrae* PB90-1 | S002287922 |
| Lentisphaerae | *Lentisphaera araneosa* HTCC2155 | S000358543 |
| Proteobacteria | *Myxococcus xanthus* DK 1622 | S004066379 |
| Nitrospinae | *Nitrospina gracilis* 3/211 | S002907032 |
| Nitrospirae | Candidatus *Nitrospira defluvii* ERS379726 | S002289961 |
| Acidobacteria | *Terriglobus saanensis* SP1PR4 | S004065355 |
| Elusimicrobia | *Endomicrobium proavitum* Rsa215 | S004460533 |
| Synergistetes | *Aminomonas paucivorans* DSM 12260 | S004070658 |
| Spirochaetes | *Turneriella parva* DSM 21527 | S004064837 |

References

1 Braun, V. & Endriss, F. Energy-coupled outer membrane transport proteins and regulatory proteins. *Biometals* **20**, 219-231 (2007).

2 Di Tommaso, P. *et al.* T-Coffee: a web server for the multiple sequence alignment of protein and RNA sequences using structural information and homology extension. *Nucleic Acids Res* **39**, W13-17 (2011).

3 Hall, T. A. BioEdit: a user-friendly biological sequence alignment editor and analysis program for Windows 95/98/NT *Nucl. Acids. Symp. Ser.* **41**, 95-98 (1999).

4 Moynie, L. *et al.* Structure and function of the PiuA and PirA siderophore-drug receptors from *Pseudomonas aeruginosa* and *Acinetobacter baumannii*. *Antimicrob Agents Chemother* **61**, e02531-02516 (2017).

5 Buchanan, S. K. *et al.* Structure of colicin I receptor bound to the R-domain of colicin Ia: implications for protein import. *EMBO J* **26**, 2594-2604 (2007).

6 Cobessi, D., Celia, H. & Pattus, F. Crystal structure at high resolution of ferric-pyochelin and its membrane receptor FptA from *Pseudomonas aeruginosa*. *J Mol Biol* **352**, 893-904 (2005).

7 Lukacik, P. *et al.* Structural engineering of a phage lysin that targets gram-negative pathogens. *Proc Natl Acad Sci U S A* **109**, 9857-9862 (2012).

8 Freed, D. M., Horanyi, P. S., Wiener, M. C. & Cafiso, D. S. Conformational exchange in a membrane transport protein is altered in protein crystals. *Biophys J* **99**, 1604-1610 (2010).

9 Chimento, D. P., Mohanty, A. K., Kadner, R. J. & Wiener, M. C. Substrate-induced transmembrane signaling in the cobalamin transporter BtuB. *Nat Struct Biol* **10**, 394-401 (2003).

10 Shultis, D. D., Purdy, M. D., Banchs, C. N. & Wiener, M. C. Outer membrane active transport: Structure of the BtuB:TonB complex. *Science* **312**, 1396-1399 (2006).

11 Ferguson, A. D., Hofmann, E., Coulton, J. W., Diederichs, K. & Welte, W. Siderophore-mediated iron transport: crystal structure of FhuA with bound lipopolysaccharide. *Science* **282**, 2215-2220 (1998).

12 Locher, K. P. *et al.* Transmembrane signaling across the ligand-gated FhuA receptor: crystal structures of free and ferrichrome-bound states reveal allosteric changes. *Cell* **95**, 771-778 (1998).

13 Brillet, K., Meksem, A., Lauber, E., Reimmann, C. & Cobessi, D. Use of an in-house approach to study the three-dimensional structures of various outer membrane proteins: structure of the alcaligin outer membrane transporter FauA from *Bordetella pertussis*. *Acta Crystallogr D Biol Crystallogr* **65**, 326-331 (2009).

14 Cobessi, D., Meksem, A. & Brillet, K. Structure of the heme/hemoglobin outer membrane receptor ShuA from *Shigella dysenteriae*: Heme binding by an induced fit mechanism. *Proteins* **78**, 286-294 (2010).

15 Krieg, S. *et al.* Heme uptake across the outer membrane as revealed by crystal structures of the receptor–hemophore complex. *Proc. Natl. Acad. Sci. USA* **106**, 1045-1050 (2009).

16 Brillet, K. *et al.* A beta strand lock exchange for signal transduction in TonB-dependent transducers on the basis of a common structural motif. *Structure* **15**, 1383-1391 (2007).

17 Greenwald, J. *et al.* FpvA bound to non-cognate pyoverdines: molecular basis of siderophore recognition by an iron transporter. *Mol. Microbiol.* **72**, 1246-1259 (2009).

18 Cobessi, D. *et al.* The crystal structure of the pyoverdine outer membrane receptor FpvA from *Pseudomonas aeruginosa* at 3.6 angstroms resolution. *J Mol Biol* **347**, 121-134 (2005).

19 White, P. *et al.* Exploitation of an iron transporter for bacterial protein antibiotic import. *Proc Natl Acad Sci U S A* **114**, 12051-12056 (2017).

20 Ferguson, A. D. *et al.* Structural basis of gating by the outer membrane transporter FecA. *Science* **295**, 1715-1719 (2002).

21 Yue, W. W., Grizot, S. & Buchanan, S. K. Structural evidence for iron-free citrate and ferric citrate binding to the TonB-dependent outer membrane transporter FecA. *J Mol Biol* **332**, 353-368 (2003).

22 Buchanan, S. K. *et al.* Crystal structure of the outer membrane active transporter FepA from *Escherichia coli*. *Nat Struct Biol* **6**, 56-63 (1999).

23 Grinter, R. *et al.* Structure of the bacterial plant-ferredoxin receptor FusA. *Nat. Commun.* **7**, 13308 (2016).

24 Brillet, K. *et al.* Pyochelin enantiomers and their outer-membrane siderophore transporters in fluorescent *Pseudomonads*: Structural bases for unique enantiospecific recognition. *J. Am. Chem. Soc.* **133**, 16503-16509 (2011).

25 Saleem, M. *et al.* Use of a molecular decoy to segregate transport from antigenicity in the FrpB iron transporter from *Neisseria meningitidis*. *PLOS ONE* **8**, e56746 (2013).

26 Calmettes, C. *et al.* The molecular mechanism of Zinc acquisition by the neisserial outer-membrane transporter ZnuD. *Nat Commun* **6**, 7996 (2015).

27 Noinaj, N. *et al.* Structural basis for iron piracy by pathogenic *Neisseria*. *Nature* **483**, 53-58 (2012).

28 Glenwright, A. J. *et al.* Structural basis for nutrient acquisition by dominant members of the human gut microbiota. *Nature* **541**, 407-411 (2017).

29 Kelley, L. A., Mezulis, S., Yates, C. M., Wass, M. N. & Sternberg, M. J. The Phyre2 web portal for protein modeling, prediction and analysis. *Nat. Proto* **10**, 845-858 (2015).

30 Hodgkin, J. & Kaiser, D. Cell-to-cell stimulation of movement in nonmotile mutants of *Myxococcus*. *Proc. Natl. Acad. Sci. USA* **74**, 2938-2942 (1977).

31 Wu, S. S., Wu, J. & Kaiser, D. The *Myxococcus xanthus pilT* locus is required for social gliding motility although pili are still produced. *Mol. Microbiol.* **23**, 109-121 (1997).

32 Hodgkin, J. & Kaiser, D. Genetics of gliding motility in *Myxococcus xanthus* (Myxobacterales): Two gene systems control movement. *Mol. Gen. Genet.* **171**, 177-191 (1979).

33 Dey, A. *et al.* Sibling rivalry in *Myxococcus xanthus* is mediated by kin recognition and a polyploid prophage. *J. Bacteriol.* **198**, 994-1004 (2016).

34 Diodati, M. E. *et al.* Nla18, a key regulatory protein required for normal growth and development of *Myxococcus xanthus*. *J Bacteriol* **188**, 1733-1743 (2006).

35 Konovalova, A., Löbach, S. & Søgaard-Andersen, L. A RelA-dependent two-tiered regulated proteolysis cascade controls synthesis of a contact-dependent intercellular signal in *Myxococcus xanthus*. *Mol Microbiol* **84**, 260-275 (2012).

36 Julien, B., Kaiser, A. D. & Garza, A. Spatial control of cell differentiation in *Myxococcus xanthus*. *Proc. Natl. Acad. Sci. USA* **97**, 9098-9103 (2000).

37 Jakovljevic, V., Leonardy, S., Hoppert, M. & Søgaard-Andersen, L. PilB and PilT are ATPases acting antagonistically in type IV pilus function in *Myxococcus xanthus*. *J. Bacteriol.* **190**, 2411-2421 (2008).

38 Karimova, G., Ullmann, A. & Ladant, D. Protein-protein interaction between *Bacillus stearothermophilus* tyrosyl-tRNA synthetase subdomains revealed by a bacterial two-hybrid system. *J. Mol. Microbiol. Biotech.* **3**, 73-82 (2001).

39 Karimova, G., Dautin, N. & Ladant, D. Interaction network among *Escherichia coli* membrane proteins involved in cell division as revealed by bacterial two-hybrid analysis. *J. Bacteriol.* **187**, 2233-2243 (2005).
